# Analysis of human resources for health strategies and policies in 5 countries in Sub-Saharan Africa, in response to GFATM and PEPFAR-funded HIV-activities

**DOI:** 10.1186/1744-8603-9-52

**Published:** 2013-10-25

**Authors:** Johann Cailhol, Isabel Craveiro, Tavares Madede, Elsie Makoa, Thubelihle Mathole, Ann Neo Parsons, Luc Van Leemput, Regien Biesma, Ruairi Brugha, Baltazar Chilundo, Uta Lehmann, Gilles Dussault, Wim Van Damme, David Sanders

**Affiliations:** 1 School of Public Health, Faculty of Community Health Sciences, University of the Western Cape, Cape Town, South Africa; 2 Unit of International Public Health and Biostatistics, Instituto de Higiene e Medicina Tropical, CMDT, WHO Collaborating Centre for Health Workforce Policy and Planning, Universidade Nova de Lisboa, Lisbon, Portugal; 3 Eduardo Mondlane University, Maputo, Mozambique; 4 Faculty of Health Sciences, National University of Lesotho, Maseru, Lesotho; 5 Department of Public Health, Institute of Tropical Medicine, Antwerp, Belgium; 6 Department of Epidemiology and Public Health Medicine, Division of Population Health Sciences, Royal College of Surgeons in Ireland, Dublin 2, Ireland

**Keywords:** Human resources for health, Sub-Saharan Africa, HRH policies, Global health initiatives, HIV, GFATM, PEPFAR, Health system strengthening

## Abstract

**Background:**

Global Health Initiatives (GHIs), aiming at reducing the impact of specific diseases such as Human Immunodeficiency Virus (HIV), have flourished since 2000. Amongst these, PEPFAR and GFATM have provided a substantial amount of funding to countries affected by HIV, predominantly for delivery of antiretroviral therapy (ARV) and prevention strategies. Since the need for additional human resources for health (HRH) was not initially considered by GHIs, countries, to allow ARV scale-up, implemented short-term HRH strategies, adapted to GHI-funding conditionality. Such strategies differed from one country to another and slowly evolved to long-term HRH policies. The processes and content of HRH policy shifts in 5 countries in Sub-Saharan Africa were examined.

**Methods:**

A multi-country study was conducted from 2007 to 2011 in 5 countries (Angola, Burundi, Lesotho, Mozambique and South Africa), to assess the impact of GHIs on the health system, using a mixed methods design. This paper focuses on the impact of GFATM and PEPFAR on HRH policies. Qualitative data consisted of semi-structured interviews undertaken at national and sub-national levels and analysis of secondary data from national reports. Data were analysed in order to extract countries’ responses to HRH challenges posed by implementation of HIV-related activities. Common themes across the 5 countries were selected and compared in light of each country context.

**Results:**

In all countries successful ARV roll-out was observed, despite HRH shortages. This was a result of mostly short-term emergency response by GHI-funded Non-Governmental Organizations (NGOs) and to a lesser extent by governments, consisting of using and increasing available HRH for HIV tasks. As challenges and limits of short-term HRH strategies were revealed and HIV became a chronic disease, the 5 countries slowly implemented mid to long-term HRH strategies, such as formalisation of pilot initiatives, increase in HRH production and mitigation of internal migration of HRH, sometimes in collaboration with GHIs.

**Conclusion:**

Sustainable HRH strengthening is a complex process, depending mostly on HRH production and retention factors, these factors being country-specific. GHIs could assist in these strategies, provided that they are flexible enough to incorporate country-specific needs in terms of funding, that they coordinate at global-level and minimise conditionality for countries.

## Background

In the early 2000s the international community responded to the Human Immunodeficiency Virus (HIV) pandemic, in addition to re-emerging Tuberculosis (TB) and on-going malaria epidemics, by creating targeted disease-specific funding mechanisms, termed Global Health Initiatives (GHIs). Among those funding for HIV control were the U.S. President’s Emergency Plan For AIDS Relief (PEPFAR), the Global Fund for AIDS, Tuberculosis and Malaria (GFATM) and the Multicountry AIDS Program of the World Bank (MAP) [1]. GHIs were defined as “*a blueprint for financing, resourcing, coordinating, and/or implementing disease control across at least several countries in more than one region in the world*” [2], pp. 74. GFATM and PEPFAR allocated most funds to antiretroviral (ARV) provision [3,4], which was the most pressing need at country-level at that time, without considering human capacity to deliver these ARVs.

Rapid expansion of HIV programmes in Sub-Saharan Africa (SSA) suddenly revealed the demand for human resources for health (HRH) in a continent already depleted of HRH [5,6]. HRH demand was not only quantitative, in terms of the scale-up of labour-intensive service-delivery to hundreds of thousands of newly diagnosed patients, but also qualitative, in terms of new skills needed to manage patients with HIV [7]. SSA was faced with a double challenge in relation to HRH, a structural deficit and an acquired shortage. The World Health Organization (WHO) had estimated that 2.3 qualified health workers *per* 1,000 population – 0.55 physicians and 1.88 nurses/midwives - were the minimum, needed to ensure the delivery of 80% of basic services [5]. This minimum threshold, which varies in relation to the epidemiological profile and the productivity of staff, was far from being achieved in most the countries in SSA at the time GHIs launched their activities.

The distorting effects of HIV funding on countries’ health systems, including effects on HRH, were being reported from soon after the launch of GHIs: PEPFAR, by channelling their funding through international implementers and GFATM, by implementing their activities through national non-governmental organizations (NGOs), were responsible for “poaching” staff from an already depleted public sector; in many sites, specific units for HIV patients were created in public facilities around a doctor-centred model of ARV provision, fragmenting the health system; and the skills needed for HIV management were initially developed in standalone programmes and in an uncoordinated fashion, mostly through in-service trainings [8-13].

Such country-level findings, which were often based on key informant interviews at the national level, brought the HRH issue to the attention of global stakeholders and became incorporated into the “health systems strengthening” global agenda. This was evidenced by a number of global initiatives on HRH which multiplied during the last decade: Joint Learning Initiative [14], World health Report [5], Global Health Workforce Alliance, Japanese International Cooperation Agency’s commitment to train additional health workers, renewal of interest in Community-Health Workers and Mid-Level Workers, WHO global plan to “treat, train and retain” HRH [15].

At country-level, a number of innovative approaches and new policies, different in nature according to the context, were also developed in response to the aggravated HRH crisis.

This paper provides an analysis of both short-term and long-term HRH responses to implementation of PEPFAR and GFATM funded activities, in 5 countries in SSA. It draws on health policy and system research, an approach born from the necessity to analyse systematically globalized and complex health systems.

## Methods

A five year multi-country study (2007–2011), funded by the European Commission was conducted in 5 countries in SSA (Angola, Burundi, Lesotho, Mozambique and South Africa), to describe and analyse the effects of GHIs on country health systems. GHIs common to all 5 countries were PEPFAR (except Burundi), MAP (except South Africa) and GFATM. MAP was excluded from the study since not all countries could collect data related to it. The research focused mainly on countries’ responses to the increased demand on HRH due to the challenges posed by GHI-funded HIV activities.

The research design was a mixed methods approach, based on a conceptual framework developed to analyse the influence of external aid on countries’ health policy and systems. Tools for quantitative and qualitative data collection were jointly developed by the multi-country team, covering the 6 building blocks of the health system [13].

Details on each country’s method for data collection and analysis are presented in Table 1. Ethical approval and permission to conduct research were obtained from relevant authorities in all countries prior to data collection. Sampling, from provinces down to facilities, was done by each country team, and finalised following consultation with relevant authorities.

**Table 1:** Methods used and details of data collection and analysis in each country

	**Angola**	**Burundi**	**Lesotho**	**Mozambique**	**SA**
Ethics approval	National and provincial health authorities	National ethics committee	Ethics committee of the Ministry of Health and Social Welfare	National bioethics committee	National, provincial and municipal government research committees, UWC ethics committee
Period of data collection	2009 April-June	2009 February- June	2008 July- 2009 February	2007 March- September; 2008	2008 September -2010 October
	2010 May	2011 March- June		2010 February-May	
	2011 June-September				
Language of data collection	Portuguese	French / Kirundi	English	Portuguese and English	English, Xhosa, Afrikaans, Zulu
Number and type of interviews	National level: Ministry of Health (minister, advisors from the Ministry of Health / PAV-MINSA “immunization program”), and offices of selected Implementing Partners (UNICEF, ONUSIDA, EU, WHO) and NGOs 11 in 2009 1 in 2010	National level: MoH officials (senior managers, HRH/ planning/ programs managers), National AIDS Council secretariat, NGOs representatives, GHIs representatives 27 in 2009 26 in 2011	National level : in-depth key informant interviews with 22 representatives of the government, bilateral and multilateral development agencies and other stakeholders at the national level	National level: 21 in 2008; Ministry of Health (MoH) officials, offices of selected Implementing Partners (WHO, UNAIDS, UNICEF, Irish Aid, PMI/CDC, World Bank, USAID, CDC, DFID) and NGOs (MONASO-network of national NGOs working on AIDS, Malaria Consortium, Health Alliance International	National level: MoH officials, SANAC, international NGO coordinators, international health agencies coordinators 19 in 2008–2009 18 in 2010
	Sub-national level: provincial government officers, NGOs, district managers, facility managers 30 in 2011	Sub-national level: provincial government officers, provincial AIDS committee, NGOs, district managers, facility managers and employees 35 in 2009 45 in 2011		Sub-national level: around 60 with provincial and district health directorates, HR managers, NGO managers, individuals in charge of health facilities and services responsible in 2010	Sub-national levels: Provincial and municipal governments, sub-provincial management levels (general manager, HR manager, finance manager) N = 105 from 2009 to 2010
					Facility and NGO level: NGO representative, health workers, facility manager N = 144
Type of documents analyzed	National health policy, national programs of Malaria, Tuberculosis and Maternal Health and national and international reports; Published literature and unpublished documents provided by key informants of MINSA, national and international NGOs, Provincial Health Department and Municipal Hospitals.	Policy and planning documents from programs and HRH unit, national health plan, proposals for GHIs	Review of Lesotho’s Round 5 Grant Score Cards Grant Performance reports and policy and planning documents from programs and HRH unit, national plan : 10-15	Policy and planning documents from National programs and HR National Directorate, national health plan, grey documents	Policy and planning documents from national department of health, grey documents, proposals for GHIs, draft policies
Quantitative sampling method (sub-national level	NA	3 provinces (2 rural and one urban, in each of which 3 or 4 facilities (NGO, private and public) were selected (14 in 2009 and 12 in 2011)	NA	NA	3 provinces, minimum of 2 districts in each, minimum of two facilities in each district at lowest level providing ART initiation. Rural/urban sampling where possible at each level
Type of quantitative data	HRH national report and national HIV program report for ART patients figures	Survey at facility level (N = 105 in 2009, N = 78 in 2011) to health workers (salary level, incentives, trainings, supervision)	NA	Surveys at facility level for HRH, infrastructures mapping, health information system, and pharmacy information; surveys for NGO mapping and district health services network	Health system trust database for HRH data;
					National ART report for ART patients number
					Surveys at facility level for HRH number and trainings, surveys for NGO mapping and district health services network
		HRH national review and national HIV program report for ART patients figures			
Analysis method (software used)	Qualitative data: Thematic analysis - analyzed manually	Qualitative data: Framework analysis using Atlas.ti for	Qualitative data: Framework analysis using Atlas.ti	Qualitative data: Thematic analysis using Nvivo and content analysis	Qualitative data: Thematic analysis both manually and using Atlas.ti
		Quantitative data: analyzed using Stata (version 8)			

Qualitative data consisted of document review and semi-structured interviews, conducted at different levels of the health system. A first group of respondents was selected according to their relevance to the topic researched and thereafter more were recruited using the snowball technique. A total of 145 interviews at national-level and 419 at sub-national level were conducted in the 5 countries. National-level interviews included key-informant interviews at national and sub-national levels and of development partners (international and local) and government officials.

Interviews were recorded whenever possible, transcribed, translated when needed and coded. Written consent form was obtained prior to each interview. Data in each country were analysed by country teams, constituted of at least 2 researchers, using thematic analysis, done either manually or with software. Annual workshops were conducted to discuss data collection challenges and preliminary findings, to allow triangulation of data and to identify relevant common themes.

Quantitative data consisted of surveys at facility level and secondary data from government information systems. Two categories of HRH in the public sector were considered for quantitative data: medical doctors and professional nurses (basic/intermediate/specialized levels). Other categories, such as pharmacists and community health workers were not homogeneous enough across countries to allow comparisons, or data were missing which precluded cross-country comparisons. Specialist doctors were merged with general medical doctors. Nurse and midwife figures were merged when possible to allow comparisons with the health worker threshold above cited [5].

We provided quantitative data on evolution of numbers of HRH in the public sector (2004–2010) and on ARV roll-out (2004–2009).

This paper first presents and compares background data on each country, followed by the short-term HRH strategies adopted and challenges they posed. The paper then examines countries’ transitions from a short-term strategy to long-term HRH policy, as HIV became a chronic disease. Short term strategies were defined as strategies implemented by local stakeholders without a formal regulatory framework, mostly via internal arrangement. Long-term policies were defined as those backed-up by an official authority and a regulatory framework, allowing standardization and sustainability. We finally discuss similarities and differences in strategies and policies across countries, in light of their context and more generally, the HRH strengthening needs and the role of GHIs.

## Results

### Country contexts

Relevant features of the 5 countries studied are presented in Table 2. All 5 countries now have democratically elected governments, though most are relatively new multi-party democracies, including South Africa and Mozambique (1994) and Burundi (2005). Burundi gained independence in the 1960s, whereas Angola and Mozambique did so in 1975. These 3 countries have experienced civil wars, Mozambique just after its independence and more recently Angola and Burundi. In terms of demography, Burundi is an outlier, with 318 inhabitants per km^2^, whereas other countries’ densities range from 15 to 71 *per* km^2^. South Africa is an upper-middle income country, Lesotho and Angola are lower-middle income countries and Mozambique and Burundi are low-income countries (World Bank classification 2009). The position of the 5 countries within this classification was correlated with the amount of Official Aid for Development as % of Gross National Income: Burundi and Mozambique are the 2 countries most dependent on external aid.

**Table 2:** Summary of socio-economic and health related indicators for the 6 countries included in the analysis

	**Angola**	**Burundi**	**Lesotho**	**Mozambique**	**South Africa**
Significant historic features	Independence 1975	Independence 1962	Independence1966	Independence: 1975	Apartheid 1948-1994
	Quarter century of civil war: 1975 - 2002	Cyclic civil wars since 1963	Several military coups with latest handover to democratic government in 1995	Civil war: 1976-1992	
		Latest: 1993-2006			
Population density/km2	15	318	71	29	41
Net ODA as% of GNI	0.3	42.3	5.4	20.8	0.4
GDP in current USD per capita	4069	163	800	428	5733
Public health expenditure, as % of total government expenditure	8	12	8	13	9
External resources for health, % of total expenditure for health	3	45	30	72	2
OOP expenditure on health, % of total expenditure for health	11	36	22	12	18
GINI coefficient (latest available)	58.6 (2000)	33.3 (2006)	52.5 (2003)	47.1 (2003)	67.4 (2006)
Human Development Index ranking 2011 (out of 187 countries)	148	185	160	184	123
HIV prevalence 15–49 years old, % (2009)	2	3.3	23.6	11.5	17.8
Number of persons affected by HIV, all ages, 2009	200,000	180,000	290,000	1,400,000	5,600,000
TB incidence, per 100,000 inhabitants, 2010	304	129	633	544	981
Number of TB cases detected, 2010	58,000	11,000	14,000	130,000	490,000
Malaria mortality rate per 100,000 inhabitants, 2008	89	39	0.1	171	0.2
Under-five mortality rate, per 1,000 live births, 2010	161	142	85	135	57
Maternal mortality ratio per 100,000 live births, 2008	610	970	530	550	410
GFATM-HIV, cumulative disbursement, as of 2011, millions USD	62.2	69.5	91.8	168.7	247.6
GFATM-malaria, disbursed, as of 2011, millions USD	62.0	55.2	0	61.6	0
GFATM-TB, disbursed, as of 2011, millions USD	10.3	9.8	10.7	12.6	0
PEPFAR, disbursed, as of 2009, millions USD ( + committed 2010)	47.7	0 (Not eligible)	96.2	1096.7	3113.4
MAP1 World Bank, committed, millions USD	21	51 (with MAP2)	5	55	0

Sources:

Socio-economic and finance indicators; World Bank database, 2009.

Health indicators: WHO global health indicators database.

Disbursements indicators: GFATM website, PEPFAR country-websites, World Bank MAP-country websites.

Human development index: UNDP.

In terms of burden of HIV, Lesotho and South Africa carry the heaviest ones, with, in 2009, an antenatal HIV prevalence of 23.6% and 17.8% respectively. South Africa’s larger population meant it had more than 5.5 million HIV-infected people in 2009 and almost half a million cases of TB diagnosed in 2010. Angola and Burundi have the lowest incidence of TB (304 and 129 cases *per* 100,000 inhabitants respectively in 2010) and HIV prevalence (3.3 and 2% respectively in 2009). Angola, Burundi and Mozambique have the worst indicators for maternal and child health.

With regard to health financing, all governments spent in 2009 from 8 to 13% of their total expenditure on the health sector; this percentage included funding from external sources. The latter represents as much as 72% for Mozambique, 45% in Burundi, and 30% in Lesotho, but only 3 and 2% for Angola and South Africa respectively. If external sources of funding are excluded, government expenditure on health would drop significantly in the 4 first countries, and would then represent less than 7% of total government expenditure.

The health system organization is typically pyramidal in all countries, with a centrally managed district-based system. In Burundi, decision-making remains very centralized at national level, especially budget allocation, since the district health system is very recent. In South Africa, the national level plays a normative role, by drafting policies and allocating funding, while provinces have considerable autonomy and exhibit variation. In Angola, the national level has a normative role and since 2007, municipal health authorities are in charge of district health management (including budget allocation) and planning. In Mozambique and in Lesotho, the decentralization process has been gradual. In Mozambique, provinces gained autonomy, while budget allocation is still centralized. All countries have HRH development policies and plans, but their level of ownership and sophistication varies: South Africa’s plan is relatively detailed with projections of HRH until 2025 [16]; Mozambique’s is clearly articulated with MDGs and aligned with other policies but lacks funding (National Plan for HRH Development 2008–2015; National Directorate of Human Resources, Ministry of Health, 2008); whereas Burundi launched its first plan only in 2010 [17]; Lesotho’s HRH policy is clearly articulated with the Health and Social Welfare Policy (2004) which ensures appropriate supply, and properly trained personnel to meet the needs of the country and also ensures career development.

**Figure 1:**
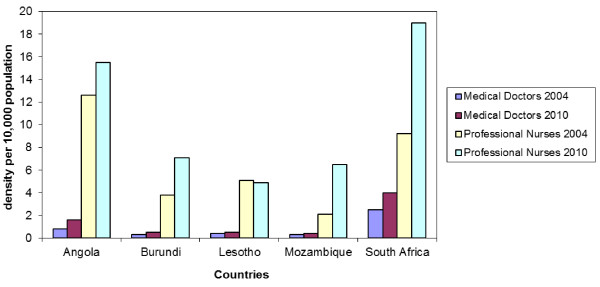
Evolution of selected HRH indicators in the public sector, in the 5 countries, between 2004 and 2010.

In terms of HRH quantity, all countries except South Africa have experienced a general HRH deficit [18]. However, these figures are aggregates, neglecting the breakdown between public and private for profit (PFP) staff. In South Africa, the PFP sector is favoured by physicians, with 59% of them working in this sector in 2010, while serving only 15% of the population [19]. In the other 4 countries, the PFP sector is not so prominent, though its assessment is difficult, since many HRH working in the public sector are in dual practice, undertaking a parallel PFP activity. If only the public sector is considered and the threshold of 2.3 health workers (0.55 doctors and 1.88 nurses and midwives *per* 1,000 population) applied, all 5 countries have unmet needs, both in 2004 and 2010 (Figure 1). The extent of unmet needs in the public sector varies, with the lowest density of physicians and professional nurses in Burundi and Mozambique, whereas Angola has a shortage of physicians and has nurses in excess (though most of them have only basic level education) when compared to the WHO threshold.

Since at least 2004, all 5 countries have been receiving funding from the 3 GHIs (GFATM, PEPFAR -except for Burundi- and the World Bank MAP -except for South Africa-).The amounts disbursed by PEPFAR greatly outweighed those of GFATM and MAP, especially in South Africa and Mozambique (Table 2), much of it for ARV roll-out. Lesotho was not a focus-country for PEPFAR and Burundi was not eligible for PEPFAR until 2011. In terms of subsequent ARV roll-out, the increase in the number of patients on ARV was dramatic in all 5 countries between 2004 and 2009 (Table 3), despite the sustained deficit in HRH (Figure 1). The majority of patients were enrolled for ARV in the public sector, except for Burundi where the majority were initially enrolled in stand-alone HIV-clinics run by local NGOs. Data from our study showed that this successful ARV roll-out, despite a HRH deficit in these countries, was a result of adaptive practices, which occurred in a similar way in all countries: initially, a short-term emergency response mostly by GHI-funded NGOs and to a lesser extent by government; then, challenges related to this emergency-type response increased and, as HIV became a chronic disease, government started to formulate and implement long-term policies, sometimes in collaboration with GHIs. This 2-step country-response is detailed below.

**Table 3:** Numbers of patients on ARV in 2004 and 2009 in the 5 countries

**Year**	**Angola**	**Burundi**	**Lesotho**	**Mozambique**	**South Africa**
2004 (unless otherwise specified)	5,357	1,200	54,237	8,010	32,895 (2005)
2009 (unless otherwise specified)	20,640	17,500 (2010)	92,773 (2008)	134,147	781,465

Sources:

Angola, Burundi, Lesotho, Mozambique: national HIV program reports.

SA: ART factsheet from national department of health.

### Short term strategies: facing the HIV emergency

#### Using the pool of available HRH for HIV tasks

##### Informal or formal task-shifting at facility-level

In all countries except Angola, task-shifting (to less skilled HRH or to lay health workers) occurred at facility-level for HIV clinical management. In South Africa, it was mainly an initiative of international NGOs, first informally by Médecins Sans Frontières (MSF), during the period of HIV denialism in the government [20], followed by GHI-funded NGOs. In Burundi, informal task-shifting was in place in rural areas since before the civil war, because of pre-existing doctors’ shortage. However, task-shifting for HIV was not considered initially. More recently, informal initiatives with follow-up of ARV patients by nurses were conducted as pilots, mostly by GFATM-supported NGOs. In Mozambique, the Ministry of Health adopted HIV task-shifting policy early on: formal non-physician clinicians pre-dated the GHIs era and have been further trained for ARV prescription; also, in some rural facilities, nurses were trained to follow-up pre-ARV patients. In Lesotho, task-shifting has always been supported by the government. Nurses in the rural areas have always worked under a written protocol by the district medical officer. This has been extended to HIV/AIDS; nurses have been trained further to initiate ARV treatment in uncomplicated cases.

Challenges: In South Africa, facility-level task-shifting was well accepted locally but remained for a while as scattered pilot projects; and its scale-up was slow, due to a lack of formal process and standardization and an active opposition from professional councils. In Burundi, its uptake was slow, mainly due to some resistance to change from professional bodies as well as from patients, since HIV was considered a complex disease. In Mozambique and Lesotho, where task-shifting was operating historically since before GHIs, its adaptation to include HIV activities was easier.

##### Allocation of HIV-tasks to specific health workers

In all countries except Angola, specific units for HIV services opened in public facilities. Separation of HIV and non-HIV activities aimed at tighter management of external funding and at training a limited number of HRH. New staff were recruited or staff formerly present were internally redeployed to work in these units. Sometimes one staff member was allocated to HIV-specific tasks within the same unit (e.g. Prevention of Mother-To-Child Transmission -PMTCT- in Maternal and Child Unit).

Challenges: In the short-term, this system allowed implementation of urgent and specific activities but proved unsustainable in the medium-term. Indeed, the numbers of specific HRH needed (clinical as well as administrative) had to be increased exponentially, in parallel to patient numbers. In Mozambique, this stand-alone approach was discontinued in 2007 and HIV services were progressively integrated into the public health sector. Also, except in South Africa, staff within these specific HIV units and in some cases staff allocated to specific HIV tasks within the same unit received supplementary stipends out of GFATM-HIV grant. These practices induced team divisions and tended to undermine mainstreaming of HIV activities.

#### Increasing the pool of HRH for HIV tasks

##### Use of retired staff (only in Lesotho)

Retired nurses are used as mentors for PMTCT (by international NGOs) - administration of that program is being moved to the government with local mentors being paid by the government. This strategy has worked because Lesotho was in dire shortage of nurses and utilized available and experienced staff. The biggest challenge was the remuneration while nurses were transferred back to government, since international NGOs paid more than the government.

##### Stand-alone GHI funded NGOs for HIV-activities

In the case of PEPFAR and GFATM, NGOs delivering HIV-care were either direct or indirect recipients of funding, as disbursement through civil society was a conditionality to get their funding [21,22]. In all countries, local NGOs were mainly funded by GFATM whereas PEPFAR implementers, who subcontracted to local NGOs, were often U.S.-based. In Burundi and Mozambique, GHI implementing agencies, separate from government, were created. In Burundi, these agencies also funded local NGOs and some of these in turn created HIV-clinics, run independently from governmental institutions. In all countries, GHI-funded NGOs offered higher salaries than the public sector, allowing them to attract more qualified staff. In Burundi, on average, GFATM-funded NGO staff was paid 2 to 3 times more than in the public sector at the time of the survey at facility-level (quantitative data, 2009). In Mozambique, Angola and Lesotho, key informants all reported that PEPFAR implementers provided much higher salaries than GFATM-funded NGOs.

Challenges: All countries faced an internal brain-drain of experienced staff from the public sector to GHI-funded NGOs, due to differences in salary level. In Burundi and Lesotho, migration was also observed from the public sector to the National AIDS Council (NAC) secretariat, where management staff received higher salaries than in the public sector.

##### HIV staff seconded to facilities - (in South Africa only)

A number of PEPFAR implementers were allowed by government to second staff to public sector HIV clinics, to help them with accreditation and to then hand over the clinic to the facility in which they were located. In one province, GFATM-funded HRH, dedicated to HIV, were placed at facility level and directly managed by the provincial department of health.

Challenges: Clinical staff, paid by PEPFAR implementers and seconded to facilities, were officially paid at the same rate as public sector staff, as a result of internal arrangement, but had higher net incomes as government benefits were not automatically deducted, incurring some resentment from the rest of the staff. The vertical management system of GFATM created confusion and undermined integration. Concerns around whether these seconded staff would be absorbed by the public sector were being raised, as GHI funding began declining.

##### Informal task-shifting at community-level

In all countries, Community Health Workers (CHWs) existed before the GHI era. In South Africa, CHWs were further recruited and trained, mostly by local NGOs subsidized by GFATM grants, to provide prevention and promotion activities on HIV at community level. In Burundi, Mozambique and Lesotho, new cadres of health workers called “health mediators”, “lay counsellors” or “expert-patients” were introduced through funding from GHIs, dedicated exclusively to HIV-related activities [23]. Their training has been provided by GHI-funded NGOs (Lesotho). After their training, they receive short-term contracts with NGOs or hospitals, working under the supervision of NGOs and receiving GHI-funded stipends.

Challenges: In contrast to implementation of task-shifting at facility-level, community-level task-shifting worked well, since it preceded the emergence of the GHIs and did not interfere with existing power and hierarchies amongst professional HRH. However, in Burundi, the curricula of the new cadres of HRH remained 'unofficial’ rendering their situation precarious. “Health mediators” were left unpaid for months, due to confusion during the transition between 2 GFATM rounds [24]. The lack of harmonization of trainings amongst different training providers was also a concern in all countries.

### HIV as a chronic disease: towards long-term policies

#### Formalisation of pilot initiatives

##### Regulatory framework for task-shifting at facility-level

Informal task-shifting was followed by attempts to legitimise expanded professional responsibilities and to standardize practices. In South Africa, the government authorized formal nurse-initiated antiretroviral therapy [25], after the STRETCH (Streamlining Tasks and Roles to Expand Treatment and Care for HIV) trial demonstrated the safety of such a policy [26]. In Burundi in 2008, a ministerial circular authorized nurses to prescribe ARV, though under the supervision of a medical doctor. In Mozambique and Lesotho, where task-shifting pre-dated GHIs, scopes of practice of non-physician clinicians and nurses were officially expanded, with HIV training provided by PEPFAR and international implementing partners (e.g. International Training & Education Centre for Health in Mozambique).

##### Official creation of new cadre of health workers

Two new cadres of health professionals were officially created in South Africa in 2008 with their job description released by national level through a circular in 2010 [27,28]. The curriculum of one of them, called clinical associates, includes also HIV management and is partially supported by PEPFAR (funding of HIV/AIDS twinning centre partnerships with US institutions). A middle-level pharmacy cadre, called pharmacists' assistants, has also been created, with some initial resistance from the pharmaceutical bodies. CHWs are recognized in South Africa as having significantly supported ARV roll-out. With the latest, far-reaching reforms in South Africa, known as 'Primary Health Care (PHC) re-engineering’, CHWs are now being integrated into so-called “ward-based PHC outreach teams”, working under the supervision of nurses and as part of the formal health system [29,30].

#### Increasing the overall quantity of HRH

##### Increase in production

In reaction to overall HRH shortages, all governments made plans to increase local production, sometimes in collaboration with GHIs. In South Africa, nurses’ colleges which had been closed down were due to reopen, as part of the new HRH plan, and production by medical schools was due to increase. In Angola, 6 public medical schools were established in 2009 in the provinces, in addition to a pre-existing private school. In Burundi, production of doctors and nurses has increased due to an increase in student intake but also with the creation of private schools, though without a clear linkage between Ministries of Health and Education.

In general, the capacity of pre-service training institutions was already overstretched before the HIV crisis, raising concerns about the quality of graduates. These production increases were sometimes supported by bilateral cooperation: in Lesotho, Irish Aid contributed to training 150 additional nurses in year 2007 while some nurses were recruited from Kenya to work in the remote areas of the country; South Africa has a bilateral agreement with Cuba, to train doctors. PEPFAR supports HRH production since 2011 (in South Africa and Mozambique through Medical Education Partnership Initiative-MEPI- and in Lesotho through Nursing Education Partnership Initiative-NEPI-) [31,32].

#### Mitigation of internal migration induced by inequalities in salaries

##### Anti-poaching agreements

South Africa was the only country where evidence of informal policy adopted by a number of NGOs to stop the poaching of HRH from the public sector was reported (in one province only). In this case, NGOs signed an agreement with provincial authorities to not recruit public sector HRH from the same province. However, it did not prevent poaching from other provinces.

##### Alignment of NGO salaries to those of public sector

As a result of significant salary inequity between GHI-funded NGOs and the public sector and the subsequent brain-drain, salary harmonisation was attempted in most countries, driven by government or NGOs. In Angola, a formal and mandatory alignment of NGO salaries with those of the public sector was introduced via a decree. As a result, migration of HRH from the public sector to NGOs was partially reversed. In South Africa, alignment occurred through informal and local agreements, with considerable variation between different local governments. However, such salary alignment was only for clinical or monitoring and evaluation M&E staff (and not management level staff): some NGOs proactively enquired about salary levels in the public sector, before setting their own scales upon recruitment. No formal agreement existed at higher level, i.e. provincial or national level. In Mozambique also, in 2006, the Ministry of Health implemented by decree a new policy on salary harmonization between public health sector and NGO staff seconded to public health facilities. As a result, personnel from NGOs are no longer seconded to facilities but give external support to the public health sector. This policy does not go beyond the staff seconded to facilities and as such does not address income inequalities between external NGOs and public health sector employees. In Lesotho, NGOs and other implementing partners still have their own salary scales, independent from the public sector.

##### Increase in public sector salaries and provision of incentives

In Burundi, the government was unable to enforce alignment of NGOs’ salaries to public sector ones, since this latter were outrageously low. In 2010, eventually, industrial action forced an increase in public HRH salary levels, which were aligned to those in neighbouring countries (i.e. Rwanda). Government also implemented a retention policy, including financial incentives to public sector HRH allocated to rural areas. Migration of HRH was then reversed at the same time as GHIs funding stagnated. In Lesotho, although salaries remained low, the government instituted incentives for nurses in the public sector. This was initially meant for nurses who worked with HIV/AIDS patients through the support of donor funding. The allowance was subsequently spread to cover all nurses including those working in hospitals. In Mozambique, the government introduced incentives for senior managers to prevent them from migrating to NGOs in response to evidence of such internal migration [33]. There was a similar policy in Burundi, where the financial top-up came out of GHI or bilateral projects. In South Africa, no evidence of GHI-funded incentives was found.

##### Redistribution of incentives at facility-level

Burundi and Lesotho, where some specific categories of staff were receiving incentives for TB or HIV activities, piloted a new policy of redistributing those incentives within the entire facility (a decision of NAC in agreement with facility-managers in Burundi, a decision of government in Lesotho). It did not resolve the issue of low salaries since it resulted in a very small amount per person.

##### Participation of GHIs in performance-based financing (Burundi only)

From 2010, GFATM-HIV started to support the national policy on improving HRH working conditions using a performance-based funding system. GFATM funding served partly to pay for performance related to HIV activities. The policy was mainly donor-driven and donors managed to persuade other significant funders to pool available funding.

## Discussion

This multi-country analysis shows that all 5 countries first adopted short term strategies in reaction to the HIV pandemic and subsequent GHI funding. Those strategies consisted mainly of using the pool of available HRH to deliver on the increased volume of HIV tasks, notably increased numbers on ARVs. These strategies included task-shifting at facility-level, staff attraction and retention using incentives, and creation of stand-alone HIV clinics. To a lesser extent, the pool of HRH was increased through creation and recruitment of new cadres of health workers for HIV, use of retired staff, and secondment of NGO staff to public sector facilities.

On the one hand, these strategies allowed a substantial increase in the number of patients on ARV within a few years and fulfilled the primary objective of HIV programs and funding institutions. However, on the other hand, these strategies generated tensions: between HIV-dedicated and general HRH staff, by creating parallel channels of management and inequities in incomes. While HIV-dedicated HRH positions were better paid, these positions were not sustainable and tasks were not always clearly defined.

Eventually, countries favoured certain short-term HRH strategies over others, with support from GHIs and other donors.

### Short-term strategies: implemented according to GHIs’ common funding conditionality and countries’ specific contexts

In all countries, funding from GHIs provided a substantial amount of money, usually with a clear conditionality related to quantitative targets achievement, to kick start the disbursement of the next financial tranche [21]. This mechanism of funding was new, placing significant pressure on country systems and accountability. This pressure contributed to the creation of parallel HIV units to enable M&E reporting to GHIs, and to allow targets to be reached quickly [9]. The necessity to quickly reach such targets, led to recruitment and training of HRH, who were retained using incentives. Recruitment however occurred only in a limited number, due to the restricted funding amount. The creation of vertical programs was seen to be justified by the sense of emergency, generated by a series of high level global policy meetings around 2001–2002 in response to the growing HIV epidemic in SSA. It probably also served to demonstrate the effectiveness of certain types of funding over others, thus fuelling pre-existing competition between agencies at global level [34]. In addition to the workload added by HIV care, the combination of preventative and curative care that GHIs funding was meant to deliver was new to public sector facilities, and GHIs proposals were an open call to NGOs and civil society participation [21]. This partly explains the extensive use of NGOs (local and international) as short-term strategies to overcome the HRH crisis, either by seconding their staff to facilities, or by creating stand-alone NGOs for HIV care. In addition to these explanations above, which influenced certain types of short-term HRH strategies over more long-term ones, each country reflected its specificity, according to its historical and political background.

In South Africa, the health system in the pre-GHI era was already sufficiently organized relatively to the 4 other countries. On the one hand, this allowed secondment of NGO staff to public sector facilities, according to a pre-defined agreement. On the other hand, the very hierarchical and managerial functioning of the South African health system did not immediately allow a clear task-shifting model to be implemented at facility-level. NGOs were the most innovative in this domain, testing several models of care in different facilities.

In Burundi, Lesotho and Mozambique, a parallel implementing agency, separate from the Ministry of Health, was created to manage funding from GHIs. Furthermore in Burundi, a separate Ministry of HIV/AIDS was created. Such separation between health and so-called HIV sectors, while facilitating the daily management in the short-term, created inequities in treatment between HIV-dedicated and general staff. Also in these 3 countries, the use of CHWs was well accepted, since rural areas have relied on them for decades, especially during times of civil war. In Angola, no task-shifting was employed at facility level, nor were HIV units created, possibly related to a relatively low prevalence of HIV in the country.

As challenges and limits of short-term HRH strategies were revealed, the 5 countries slowly implemented mid to long-term HRH strategies, such as formalisation of pilot initiatives (new cadre of health workers, task-shifting), increase in HRH production, and mitigation of internal migration of HRH, by increasing public HRH salaries or by imposing salary alignment on NGOs.

### The move of countries towards Health System Strengthening (HSS), including HRH strengthening

Content of HRH strengthening policy differed, according to, *inter alia,* countries’ financial and political resources and to their resultant degree of dependence on external donors. Thus, South Africa and Angola could more easily create new cadres of health workers since these governments had sufficient funds to devote to indigenous training institutions and to increase production of HRH. Some countries, such as Burundi, did not have the negotiation space to impose a code of conduct on NGOs (i.e. to set a salary scale). Such policy space depends significantly on the extent of reliance on external donors, including NGOs. Burundi preferred to increase public sector salaries instead, which was already a pressing issue. South Africa managed to impose an anti-poaching agreement on NGOs and both Angola and South Africa managed to force NGOs to align their salaries with public sector salaries. The Mozambique government forced to align salaries of NGO staff seconded to public sector facilities with those in the public sector, but did not have any control over salaries of NGO staff *per se*.

Countries also implemented HRH strengthening policies, independently of GHIs: in Angola, South Africa and Burundi, governments started to sign bilateral agreements with countries which have a surplus of HRH, such as Cuba (for South Africa, though this pre-dated GHIs), China and Egypt (Burundi). In all countries except Angola, governments implemented incentive schemes for rural areas and sometimes retention schemes for undersubscribed specialties, independently of GHIs. In South Africa, the national department of health introduced increased remuneration to some categories of public sector HRH to reduce external or internal migration ('occupational specific dispensation’). Medical interns are also required to perform community service for 2 years in rural areas. In Mozambique, health workers working in remote rural areas are allocated an “isolation subsidy” which increases with remoteness of the placement. In Lesotho, to increase rural coverage, public sector nurses are mandatorily sent to rural areas for a period of 2 years (national policy in place since before GHIs). Also, some faith-based organizations, mostly located in rural areas, provided lower salaries than government and hence are being subsidized by government. The government, with donor support, recruited nurses from Kenya and Zimbabwe to work in some of the health facilities in the remote areas. Since 2006, in Burundi, medical doctors need to spend a 2-year mandatory period in the public sector, mostly in rural areas, if they wish to specialise.

### What were the drivers for changes in GHI policies?

GHIs and governments increased their collaboration and GHIs reshuffled their scope of activities in order to fit the HRH strategies at country-level, following the Paris declaration and acknowledging the need for aid effectiveness and increased coordination. GHIs started to take HRH pre-service trainings into consideration, although production was not initially their concern. PEPFAR for instance launched the MEPI and NEPI in 2010, two programs aiming at strengthening pre-service training of HRH [35].

Whether these changes were induced by repeated criticisms of GHI’s targets which were predominantly aligned to short-term objectives, such as short-courses and in-service trainings, has been a subject of debate [36]. Attribution to one or other cause is not possible and these changes were probably a result of a mixture of events and influences: GHIs were evaluated through a pre-set mechanism (i.e. 5-year GFATM evaluations for GFATM) [21], and further analysed via independent or commissioned research; many of them emphasized the fragmenting effects GHIs were having on the health system and in particular on HRH [9,11,13,37-39]; external advocacy towards more focus on HSS and against “AIDS exceptionalism” also grew, through conferences and publications; a global HSS debate was complemented by a focus on long-term HRH production [40].

As countries’ responses to HRH challenges were reported to GHIs, some of these, such as GFATM and Global Alliance for Vaccines and Immunization (GAVI), with a participative approach, allowed criticisms to be partly addressed in a timely manner: GFATM in 2005 introduced a HSS section to their funding application and GAVI-HSS was launched in 2006 in pilot countries [41]. Other GHIs, such as PEPFAR, more rigid in its functioning and administration, also took into account such criticisms, but at a slower pace and in a more contained way [22,42]. The slow shift to more sustainable, indigenous responses towards HSS and especially HRH strengthening seems therefore a result of a dialectical relationship between GHI-driven initiatives and country responses, while the shift has also been impelled by other objective changes (e.g. availability of generic drugs). Reduction in global funding is also certainly forcing countries and GHIs to invest more in long-term action, such as pre-service training, better coordination of training, collaboration across sectors through partnerships or across programs (TB-HIV, HIV-reproductive health) and equity in salaries.

### HSS and HRH strengthening: what’s in the name?

While GHIs should be commended on their genuine efforts to improve countries’ health system, whether these HSS plans are really strengthening or just supporting the health system is a matter of debate [43]. Also, an ideological struggle seems to have arisen around whether GHIs have *de facto* strengthened health systems or had the opposite effect [44-48]. Answers to these questions will influence future HSS directions for GHIs and countries and caution is needed in interpreting them, since answers might differ depending on who the assessor is and what meaning is given to “HSS”. Suggestions for a new orientation of GHIs have started to emerge based on accumulated country-based experience and other complex factors [49].

GFATM and GAVI have both included in their funding programs a stream for HSS, but results have been mixed: GFATM interrupted its HSS program a year after its launch, due possibly to a lack of clear definition of HSS [50]. No clear objective for HSS has been set and each program has been left to strengthen its own system. GAVI-HSS programs have not taken into account sustainability: for example in Burundi, the GAVI-HSS funds were used *inter alia* to pay for fuel and salaries for drivers, in order to transport pregnant women to referral hospitals. Also, initially, an item for HRH capacity strengthening existed in all GFATM proposals, but these were mainly interpreted as in-service training by recipients or used to hire extra staff, without a clear sustainability plan. Some of these HRH are now left without a secure position since GHI funding has shrunk.

Similarly, the MEPI and NEPI are projected as examples of PEPFAR platforms for strengthening the sustainability of HRH in the countries [22] though the majority of funding is still clearly earmarked for expanding HIV management and HIV research capacity [31]. The non-coordination between GHIs at global level, impacting negatively coordination at country level, has also emerged as a common feature to all countries, in our example of HRH strategies. In an attempt to respond in a coordinated way to health system challenges, a project to develop a joint HSS funding platform between the World Bank, GAVI and GFATM was launched in 2009 [51,52]. However, the project has stalled, showing, *inter alia,* the extent to which an agreement on HSS objectives and visions is difficult to obtain [53].

Our study has some limitations.

Qualitative data were collected in a cross-sectional,manner with different time-periods (Table 1), rising issue of temporal bias while comparing findings.

Quantitative data proved difficult or impossible to compare, since HRH categories were not the same across countries, and data were not consistently available or complete for the same time periods. The numbers of HRH specific to HIV were not available; nor were the distribution of HRH according to NGOs/private/public sector. Hence, quantitative data were provided solely for an illustrative purpose and do not serve as a basis for a rigorous statistical comparison. Information on non-response rates to requests for interviews was not available consistently across the 5 countries and was therefore not presented. This could have contributed to an ascertainment bias.

Some important institutions such as professional councils were not interviewed at first, or, even when solicited, did not reply (Health Professionals Council in South Africa). This constitutes a limitation given the role that professional councils play in HRH policies.

Particular policies adopted to counteract a specific effect induced by GHIs were difficult to distinguish from more general policies adopted by the government to address the overall HRH crisis, which pre-dated GHIs.

Finally, given the open nature of the health system, attribution of changes to the influence of GHIs or activists/researchers or countries is an impossible task (and probably not useful). The truth probably lies somewhere between these different interpretations, where stream of influences mix with experiences and beliefs and create a general direction that actors tend to follow and refine through compromise and consensus. This last point refers directly to the inherent complexity of the field of health policy and system research, constantly influenced by political and social dynamics [54].

Despite these limitations, we believe that this study provides a unique insight into the complex and slow process of policy shift which has occurred in five countries, between 2007 and 2011, using the example of HRH policies and their evolution in response to GHIs and related HIV activities. Collectively, the findings point to some consistent patterns and effects of GHIs on HRH policies and the workforce, as well as some context-specific differences, across these five southern African countries.

## Conclusion

This cross-country paper has shown the differences in countries’ strategies, in response to a common challenge, illustrating the extent to which factors inherent to countries are influential. These findings provide further evidence of the importance of country led policies in HSS [55]. Countries are best positioned to assume responsibility on how to best use the global funding available for the purpose of HSS. HSS proposals should be scrutinized carefully according to each country’s context and broader factors, since the “one size fits all” strategy has proven its inefficiency and sometimes its counter-effectiveness in the long-run. A preliminary impact assessment might be one mechanism, to anticipate any unexpected outcome due to a particular type of funding/policies on the general health system [56]. There is also an urgent need to better define what exactly “health system strengthening” means for donors, for recipients, but most importantly for health system users, i.e. for patients.

## Competing interests

The authors declare that they have no competing interests.

## Authors’ contributions

AP, TM, EM, RB, JC, IC, TM, BC carried out part of the fieldwork. DS, BC, WVD, RB, GD, UL participated in the study conception and in the design of the study. JC drafted the manuscript. JC, AP, WVD, LVL, TM, EM, RB, TM, BC, GD, RB, IC, DS, UL participated to data analysis, interpretation and edited the manuscript. All authors read and approved the final manuscript.
